# ADHD, comorbid disorders and psychosocial functioning: How representative is a child cohort study? Findings from a national patient registry

**DOI:** 10.1186/s12888-017-1204-7

**Published:** 2017-01-17

**Authors:** Beate Oerbeck, Kristin Romvig Overgaard, Stian Thoresen Aspenes, Are Hugo Pripp, Marianne Mordre, Heidi Aase, Ted Reichborn-Kjennerud, Pal Zeiner

**Affiliations:** 1 Division of Mental Health and Addiction, Oslo University Hospital, Pb. 4959 Nydalen, 0424 Oslo, Norway; 2 The Norwegian Patient Register, Trondheim, Norway; 3 Oslo University Hospital, Oslo Centre of Biostatistics and Epidemiology, Oslo, Norway; 4 Division of Mental Health, Norwegian Institute of Public Health, Oslo, Norway; 5 Institute of Clinical Medicine, University of Oslo, Oslo, Norway

**Keywords:** ADHD, Child psychiatry, National patient registry, MoBa, Multiaxial international classification of Diseases, ICD-10

## Abstract

**Background:**

Cohort studies often report findings on children with Attention Deficit Hyperactivity Disorder (ADHD) but may be biased by self-selection. The representativeness of cohort studies needs to be investigated to determine whether their findings can be generalised to the general child population. The aim of the present study was to examine the representativeness of child ADHD in the Norwegian Mother and Child Cohort Study (MoBa).

**Methods:**

The study population was children born between January 1, 2000 and December 31, 2008 registered with hyperkinetic disorders (hereafter ADHD) in the Norwegian Patient Registry during the years 2008–2013, and two groups of children with ADHD were identified in: 1. MoBa and 2. The general child population. We used the multiaxial International Classification of Diseases (ICD-10) and compared the proportions of comorbid disorders (axes I–III), abnormal psychosocial situations (axis V) and child global functioning (axis VI) between these two groups. We also compared the relative differences in the multiaxial classifications for boys and girls and for children with/without axis I comorbidity, respectively in these two groups of children with ADHD.

**Results:**

A total of 11 119 children were registered with ADHD, with significantly fewer in MoBa (1.45%) than the general child population (2.11%), *p* < 0.0001. The proportions of comorbid axis I, II, and III disorders were low, with no significant group differences. Compared with the general child population with ADHD, children with ADHD in MoBa were registered with fewer abnormal psychosocial situations (axis V: t = 7.63, *p* < .0001; d = -.18) and better child global functioning (axis VI: t = 7.93, *p* < 0.0001; d = .17). When analysing relative differences in the two groups, essentially the same patterns were found for boys and girls and for children with/without axis I comorbidity.

**Conclusions:**

Self-selection was found to affect the proportions of ADHD, psychosocial adversity and child global functioning in the cohort. However, the differences from the general population were small. This indicates that studies on ADHD and multiaxial classifications in MoBa, as well as other cohort studies with similar self-selection biases, may have reasonable generalisability to the general child population.

## Background

Attention Deficit Hyperactivity Disorder (ADHD) is a heritable, neurodevelopmental disorder with childhood onset, characterized by persistence of hyperactivity, impulsivity and attention deficit [1, 2]. The worldwide prevalence rate of childhood ADHD has been estimated to 3.40–5.29% in meta-analyses [3, 4]. Estimates indicate that boys out-represent girls by 2:1 to 9:1 depending on the ADHD subtype, and the setting, children referred to Child and Adolescent Mental Health Services (CAMHS) are more likely to be boys [5]. ADHD constitutes a major cause behind referral and follow-up in the CAMHS [6–8] and was the most common child psychiatric diagnosis (29%) in the Norwegian Patient Registry [8].

In children with ADHD, comorbid disorders are more common than not; prevalence rates of 67% and 69% were reported in two large clinical European and American multisite studies [9, 10]. Comorbid disruptive behaviour disorders are among the most prevalent in both population-based and clinical studies [9–17], and approximately half the children with ADHD have co-existing Oppositional Defiant Disorder [2]. Learning disabilities are frequent in a substantial minority, although rates vary considerably according to the definition used [18, 19]. Two review studies reported mean comorbid learning disabilities of 31% and 45% in children with ADHD, respectively [20, 21]. ADHD has been found to be associated with reduced psychosocial functioning in children both with and without learning disabilities [19] and in children with multiple comorbid psychiatric disorders [9]. Furthermore, reduced psychosocial functioning in the family was found to be more pronounced in children with co-occurring disorders [22]. In a European multisite study (*n* = 1478), similar rates of psychiatric disorders were found in boys and girls when assessed by clinicians, although parents described more emotional symptoms in girls [23]. The gender distribution of referred children varied considerably in the included sites, but no significant gender differences were found in the received treatment, or the time interval between seeking treatment and ADHD diagnosis.

Population studies are designed to detect genetic- and environmental causes of disease, and frequently present findings about ADHD [24–26]. However, participation rates in population studies are often low [27–29]. When studying childhood ADHD, there is increased risk of under-representation, because of a threefold risk of nonparticipation by people with mental disorders [30]. One study found child ADHD to be twice as prevalent among nonparticipants as among participants [31]. Furthermore, ADHD is a familial disorder [1] associated with socio-economic disadvantages (i.e., low parental educational levels and income, single parenthood) [32]. These psychosocial factors are associated with self-selection and attrition in longitudinal studies, and selective drop-outs of participants have been found to lower prevalence-rates [33]. Consequently, there is uncertainty about the extent to which participants in population studies are representative of children with ADHD in the general population. Earlier studies have investigated the possible consequences of nonparticipation using selected exposure-outcome associations around the time of birth. In spite of lower prevalence rates, they found no substantial relative group differences in the selected exposure-outcome associations, suggesting that these associations are not biased by self-selection [27, 34]. However, these studies did not investigate associations between clinical outcomes commonly under scrutiny in the ADHD literature: comorbidity of psychiatric disorders, learning disabilities, psychosocial – and global functioning.

The Norwegian Mother and Child Cohort Study (MoBa), involving about 110 000 children followed from pregnancy onwards, represents a unique opportunity to investigate risk factors, developmental trajectories and clinical features of mental disorders such as ADHD [35]. However, in line with other population studies [36–38], MoBa participants have been found to be somewhat better educated than the rest of the population, with risk groups such as young mothers and mothers living alone under-represented [34].

The present study reports on data from a nationwide patient registry using the Multiaxial Classification of Child and Adolescent Psychiatric Disorders [39, 40] on children diagnosed with ADHD reported by CAMHS. The strengths of register studies are that they reflect the daily clinical work with child disorders diagnosed only when associated with impairments in need of referral to CAMHS [10] and the registration of multiaxial classifications. The multiaxial classifications include six axes (I–VI) I: Clinical psychiatric disorders, II: Specific disorders of psychosocial development; Learning disabilities, III: Intellectual disabilities, IV: Medical conditions, V: The associated abnormal psychosocial situations [41] and VI: Children’s Global Assessment Scale; CGAS [42], range 0–100, higher values imply better functioning.

To our knowledge, the present study is the first to use information from all axes on the Multiaxial Classification of Child and Adolescent Psychiatric Disorders [40] in children with ADHD to compare proportions of these classifications between a large cohort study and the general child population. The present study adds to the previous literature by investigating these broad clinical assessments at a later age, years after pregnancy.

The aim of the present study was to examine the representativeness of child ADHD in the MoBa by using these multiaxial classifications, as reported to the Norwegian Patient Registry from CAMHS, to:

1. a. Compare the proportions of ADHD in MoBa and the general child population,
  b. Compare the proportions of comorbid disorders, abnormal psychosocial situations and child global functioning in these two ADHD groups.
2. Investigate the relative differences in multiaxial classifications for boys and girls and for children with/without axis I comorbidity, respectively, in these two ADHD groups.

Based on previous research, we hypothesized that

1. There would be a lower proportion of ADHD in MoBa than in the general child population, as well as better psychosocial- and global functioning for children with ADHD in MoBa; and
2. There would be few relative differences in multiaxial classifications for boys and girls, and for children with/without axis I comorbidity in the two ADHD groups.

## Methods

### Participants

The present study included all Norwegian children born between January 1, 2000 and December 31, 2008 that were registered with a diagnosis of ADHD reported by CAMHS to the National Patient Registry (NPR) between January 1, 2008 and December 31, 2013. In Norway, health services use the International Classification of Diseases, 10th ed. [39], with the diagnosis Hyperkinetic Disorder (code F90 or F98.8) which corresponds to the diagnosis ADHD in the Diagnostic and statistical manual of mental disorders; DSM-5 [2]. We included children in inpatient- and/or outpatient care in the CAMHS that had at least one registration of ADHD. Two groups were defined: 1. Children with ADHD in MoBa and 2. Children with ADHD in the general child population.

There were 524 726 children born in Norway in the defined time period (269 733 boys and 255 993 girls) [43], 110 230 children of whom were registered as participants in MoBa (56 533 boys, 53 697 girls). MoBa (https://www.fhi.no/studier/moba/) is a prospective population-based cohort study conducted by the Norwegian Institute of Public Health [35, 44]. Of the 277 702 pregnant women invited to take part in the study during the 10-year enrollment period, 41% agreed to participate [34, 35, 44].

The Norwegian Patient Registry (NPR) is a national health-care registry that receives patient-data reported from all the specialized mental health care services in Norway, including the CAMHS. According to the Norwegian Health Register Act and the NPR-regulation passed by the Norwegian parliament in 2007 it is mandatory for the specialized health-care services to report health-care data to the NPR. Consequently, individual-level health data are available from 2008 onward. The personal identification numbers are stored in encrypted form in the NPR. NPR receives diagnoses from CAMHS in accordance with the Multiaxial Classification of Child and Adolescent Psychiatric Disorders [39], which includes six axes; I: Clinical psychiatric disorders, II: Specific disorders of psychosocial development; Learning disabilities, III: Intellectual disabilities, IV: Medical conditions, V: Associated abnormal psychosocial situations, where every category is coded as present or absent, and an overall score of the number of different psychosocial adversities can be made [41] and VI: Children’s Global Assessment Scale; CGAS [42]. CGAS is a clinician-rated tool used in both research and clinical settings to indicate the lowest overall level of the child’s psychosocial functioning (at home, at school, with peers). CGAS is divided into 10-point intervals, with a description of the child’s level of functioning for each interval. Moderate inter-rater reliability was found when used in a clinical setting with untrained raters [45].

### Measures

For the present study, identification of the MoBa participants in the NPR was submitted to the co-author employed at the NPR, through the personal identification number. The same co-author extracted descriptive NPR data (see below) and the multiaxial classification data for children with ADHD in MoBa and in the general population. The NPR provided anonymous information from the registry to the current group of authors.

This information includes descriptive data in the form of gender, age at first CAMHS-contact and at first ADHD diagnosis, number of days and contacts in CAMHS and the multiaxial classification data: Comorbid child psychiatric disorders (axis I), learning- and intellectual disabilities (axes II and III), epilepsy (axis IV), the associated abnormal psychosocial situations (axis V), see Table 1. Summarized scores were computed for whether any of the comorbid axes I and II disorders (respectively) were present at least once. The total number of registered associated abnormal psychosocial situations comprised a computed axis V score. The child’s lowest CGAS score at the time of the ADHD-diagnosis (+/–3 months) = axis VI. (See Appendix for detailed diagnostic codes used to compute sum-scores on axes I, II and V).

**Table 1:** NPR-descriptives and multiaxial classifications in children with ADHD in MoBa (*n* = 1595) and the general child population; All (*n* = 11 119)

NPR-descriptives	Mean (sd) MoBa	Mean (sd) All	Statistics
Age (years) at first contact in CAMHS	7.07 (1.84)	7.64 (1.97)	t = -12.3, df = 1594, *p* < 0.0001, d = -.30^(a)^
Age (years) at first ADHD diagnosis in CAMHS	7.87 (1.76)	8.38 (1.96)	t = -.11.5, df =1594, *p* < 0.0001, d = -.27^(a)^
Number of days in CAMHS	858 (566)	949 (604)	*p* = 0.85^(b)^
Number of contacts in CAMHS	38 (40)	44 (51)	*p* = 0.03, d = -.13^(b)^
Multiaxial classifications	% MoBa	% All	
Axis I, psychiatric disorders	29.5%	29.5%	z = 0, *p* = 1.0^(c)^
Axis II, learning disabilities	19.3%	20.4%	z = 1.09, *p* = 0.28^(c)^
Axis III, intellectual disabilities (F70–F79)	1.3%	1.8%	z = 1.50, *p* = 0.13^(c)^
Axis IV, epilepsy (G40)	1.3%	1.3%	z = 0, *p* = 1.0^(c)^
	Mean (sd) MoBa	Mean (sd) All	
Axis V, abnormal psychosocial situations (sum-score)	0.64 (0.89)	0.81 (1.01)	t = 7.63, *p* < 0.001, d = -.18^(a)^
Axis VI, CGAS	55.0 (13.1)	52.4 (17.19	t = 7.93, *p* < 0.001, d = .17^(a)^

*NPR* Norwegian patient registry, *MoBa* the Norwegian Mother and Child Cohort Study, *CAMHS* Child and Adolescent Mental Health Clinics, *CGAS* Children’s Global Assessment Scale. ^(a)^ One sample *t*-test ^(b)^ Wilcoxon ^(c)^ Binominal test for categorical variables

### Statistics

The characteristics of the two groups of children registered with ADHD in MoBa and the general population are given as number of participants, proportions (%), means, and Cohen’s d, as appropriate. The gender distributions were estimated as odds ratios. The two ADHD groups were compared by using one sample binomial test for categorical variables, and one sample *t*-test (mean) or one sample Wilcoxon (median) for continuous variables, as appropriate, thereby accounting for the inter-dependency between children with ADHD in the MoBa and in the general child population.

We also compared the relative differences in the multiaxial classifications for boys and girls and for children with/without axis I comorbidity, respectively in these two groups of children with ADHD. For categorical variables, we used the ratio of relative frequencies where ratios below 1 indicated under-representation of the characteristic in MoBa, whereas a ratio above 1 indicated over-representation. For continuous variables, we used a one-sample *t*-test, and Cohen’s d expressed the magnitude and direction of differences. (Because of a low number of registrations, we could not analyse data from axis IV when investigating the relative differences).

## Results

Among the 524 726 Norwegian children born between January 1, 2000 and December 31, 2008, a total of 11 119 children (2.11%) were registered in the NPR with an ADHD diagnosis between January 1, 2008 and December 31, 2013, significantly more than among children in the MoBa, where 1.45% (1595/110 230) were registered with ADHD (*p* < 0.0001).

Boys had higher odds ratio (OR) for having ADHD than girls (OR = 2.79, *p* < 0.001) in the general child population, and in the MoBa (OR = 2.78, *p* < 0.001).

The MoBa participants were approximately 0.6 years younger at first contact with CAMHS, and 0.5 years younger when first registered with an ADHD diagnosis compared with the general child population (*p* < 0.0001).

Table 1 presents ADHD group comparisons using mean sum scores for axes I, II, and V, while the Appendix presents more detailed diagnostic data used to compute these sum scores.

Comorbid psychiatric disorders and learning disabilities were found in approximately 30% and 20%, respectively of children with ADHD in both MoBa and the general child population. There were no significant group differences regarding intellectual disabilities (axis III) or epilepsy (axis IV). Abnormal psychosocial situations registered on axis V were significantly less frequent in children with ADHD in MoBa compared to children with ADHD in the general child population, and child global functioning was significantly better in MoBa, with effect sizes < .30 (see Table 1). See Appendix for a detailed overview of the single international classification codes for axes I, II and V.

Tables 2 and 3 present the relative differences in multiaxial classifications for boys and girls and for children with/without axis I comorbidity, respectively, in the two ADHD groups. These results were essentially the same as those previously presented for all children with ADHD in MoBa and the general child population (Table 1); fewer abnormal psychosocial situations (axis V), and better child global functioning (axis VI) in MoBa participants, with effect sizes < .26.

**Table 2:** Comparing relative differences for boys and girls in the multiaxial classifications in the two groups of children with ADHD; MoBa and the general child population (All)

	MoBa	All	Statistics
	*N* ^(1)^ (%)	*N* ^(2)^ (%)	Ratio of relative frequencies (95% CI), *p,* d
With axis I comorbidity			
-girls	100 (24.4)	701 (24.5)	1.00 (0.84–1.20), *p* = 0.98
-boys	370 (31.2)	2576 (31.2)	1.01 (0.92–1.10), *p* = 0.88
With axis II comorbidity			
-girls	81 (19.8)	562 (19.6)	1.01 (0.82–1.25), *p* = 0.91
-boys	227 (19.2)	1704 (20.6)	0.93 (0.82–1.06), *p* = 0.28
With axis III comorbidity			
-girls	3 (0.7)	55 (1.9)	0.38 (0.12–1.22), *p* = 0.10
-boys	18 (1.5)	140 (1.7)	0.90 (0.55–1.47), *p* = 0.68
Axis V scores	Mean (SD)	Mean (SD)	
-girls	0.56 (0.79)	0.79 (0.98)	t = 4.55, *p* < 0.0001, d = -.26
-boys	0.66 (0.92)	0.82 (1.02)	t = 5.11, *p* < 0.0001, d = -.21
Axis VI scores			
-girls	55.8 (13.2)	54.2 (16.6)	t = -1.87, *p* = 0.06, d = .11
-boys	54.7 (12.9)	51.7 (17.3)	t = -5.75, *p* < 0.0001, d = .20

^1^ *MoBa*; the Norwegian Mother and Child Cohort Study: Total number of girls = 410, and boys = 1185, ^2^All: Total number of girls = 2865, and boys = 8254

**Table 3:** Comparing relative differences for children with and without axis I comorbidity in the multiaxial classifications in the two groups in the two groups of children with ADHD; MoBa and the general child population (All)

	MoBa	All	Statistics
	*N* ^(a)^ (%)	*N* ^(b)^ (%)	Ratio of relative frequencies (95% CI) *p,* d
With axis II comorbidity			
-with axis I comorbididity	104 (22.1)	666 (20.3)	1.09 (0.91–1.31), *p = 0.36*
-without axis I comorbidity	204 (18.1)	1600 (20.4)	0.89 (0.78–1.01), *p = 0.08*
With axis III comorbidity			
-with axis I comorbidity	470 (2.1)	3277 (2.4)	0.88 (0.46–1.69), *p = 0.71*
-without axis I comorbidity	11 (1.0)	116 (1.5)	0.66 (0.36–1.22), *p = 0.19*
Axis V scores	Mean (SD)	Mean (SD)	
-with axis I comorbidity	.94 (1.1)	1.17 (1.21)	t = 3.89, *p = 0.0001*, d = -.20
-without axix I comorbidity	.51 (.75)	.66 (.88)	t = 5.44, *p < 0.0001,* d = -.18
Axis VI scores			
-with axis I comorbidity	51.0 (15.3)	48.2 (18.3)	t = -3.16, *p = 0.002,* d = .17
-without axis I comorbidity	56.7 (11.7)	54.2 (16.2)	t = -4.99, *p < 0.0001,* d = .18

*MoBa* the Norwegian Mother and Child Cohort Study; ^a^MoBa: Total number with axis I-comorbidity = 470, without = 1125, ^b^All: Total number with axis I-comorbidity =3277, without = 7842

## Discussion

The present study addressed the representativeness of registered ADHD in a large population- based cohort study. We know of no other studies that have investigated representativeness by comparing clinically registered ADHD and multiaxial classifications in a cohort with those in the general population. However, previous studies have estimated bias due to nonparticipation [27, 34] and loss to follow-up [28] using pre- and perinatal exposures and a variety of outcomes. The biases in these studies were interpreted as modest for the investigated medical factors. In a MoBa study of NPR registered Autism Spectrum Disorders, self-selection bias was investigated by comparing selected pre- and perinatal exposures to Autism Spectrum Disorders in the cohort and the general child population [46]. Despite the under- and over-representation of several exposures in the cohort, all effect estimates of exposure-outcome associations deviated less than 16% from those estimated in the population. The authors concluded that self-selection did not appear to compromise validity of exposure-outcome associations in the study of Autism Spectrum Disorders in MoBa [46].

Despite differences in methodology between our study and the previous ones, our results are consistent with the overall pattern in these studies, as we found small differences in the multiaxial classifications between children with ADHD in the cohort and children with ADHD in the general population. This indicates that the findings within the MoBa-population may be generalisable on these terms.

The overall 2.11% of Norwegian children registered with ADHD is relatively similar to prevalence findings from other Nordic countries [47]. Earlier studies have shown that the variability in ADHD prevalence estimates was mostly explained by methodological characteristics of the studies [3]. The requirement of impairment, and the use of clinicians as informants lower these estimates [4, 48, 49]. In the present study, we may assume that the children were impaired as they were patients in the CAMHS. As hypothesized, we found a significantly lower proportion of ADHD diagnoses in the cohort (1.45%) than in the general child population (2.11%), suggesting self-selection, in line with other population studies reporting significantly fewer children with both ADHD [50] and Autism Spectrum Disorders [46] among study participants compared to nonparticipants.

The proportion of comorbid psychiatric disorders (axis I) found in the present study (30%) was lower than the 52% [10] and 76.7% [17] reported in two Nordic cohort studies. There is no obvious explanation for the lower proportions in the present study, but one contributing factor could be the wider age range in the other Nordic studies, which included children up to 17 years [10] and 20 years [17] compared with our upper age limit of 13 years. We may speculate whether the lower proportions of comorbid psychiatric disorders also reflect an under-reporting of clinically significant comorbidity from the clinicians to the patient registries [51]. Presumably, clinicians may be prone to registering the most impairing diagnosis only, as is often found in studies from CAMHS [52–54]. However, the overall similar proportions of comorbid axes I–III classifications (psychiatric disorders, learning- and intellectual disabilities) in MoBa and the general child population strengthen the representativeness of MoBa, as the registry data was independently collected and available for both groups of children with ADHD.

As hypothesized, we found fewer abnormal psychosocial situations (axis V) in children with ADHD in MoBa than in the general child population. This is in accordance with studies reporting that the frequency of clinical ADHD is elevated in socio-economically disadvantaged groups [55, 56] with more family problems [57, 58] and that socio-economic disadvantages may be a barrier to participation in studies [29, 30]. The better child global functioning (axis VI, CGAS) in MoBa-participants is consistent with an adult study, reporting less functional impairment in study participants than in a community sample [59]. Both ADHD-groups in the present study had mean CGAS scores indicating moderate child global impairment (52.4 and 55), similar to that of the clinical, multisite ADORE study on children with ADHD (*n* = 14 825), which reported a mean CGAS of 55 [60]. However, the slightly better CGAS in children with ADHD in MoBa (Table 1) could be partly explained by these children having fewer consultations in CAMHS (Table 1).

That children with ADHD in MoBa were significantly younger at first contact with CAMHS (and when first registered with ADHD) could be related to their better-educated parents seeking help at an early age [34]. We consider this to be one possible explanation, as received treatment has been found to be associated with higher socio-economic status, despite Norway being a highly egalitarian country, with a free, universal health-care system [61].

Earlier studies on the representativeness of cohort studies quantified bias by also comparing relative ratios (within group associations) using registry data available for both the cohorts and the general child populations [28, 46]. As this method has been regarded as a direct measure of generalisability of cohort study results [28, 46], we used a corresponding method by investigating the relative differences in multiaxial classifications within the two groups of children with ADHD. Inspection of crude numbers and effect sizes of the relative differences for boys and girls and for children with/without axis I comorbidity (Tables 2 and 3), show that they are essentially the same as those found for all children with ADHD in MoBa and the general child population (Table 1), thereby supporting the generalisability of our findings.

### Strengths and limitations

A strength of the present study is the use of the a national patient registry which as far as all data are reported as obliged, includes all patients treated within CAMHS in Norway and provides valuable information about ADHD in CAMHS and may be considered representative of clinical practice.

Our study had several limitations. First, we only included data reported to the NPR as registered by clinicians in CAMHS, thus not children diagnosed only within primary care. Second, low rates of comorbid disorders in children with ADHD may have resulted in underestimated group differences. Third, we had a follow-up period of six years and only children up until age 13 years were included. Finally, we do not know the reliability and validity of the clinically registered diagnoses.

## Conclusions

Self-selection was found to affect the proportions of ADHD, psychosocial adversity and child global functioning in the cohort. However, the differences from the general population were small. This supports that studies on ADHD and multiaxial classifications in MoBa, as well as in other cohort studies with similar self-selection biases, may have reasonable generalisability to the general child population.

## Appendix

**Table 4:** Detailed NPR-registered International Classification of Diseases codes (ICD-10) on axes I, II and V in children with ADHD; comparing MoBa participants (*n* = 1595) and the general child population (All) (*n* = 11 119)

Axis I, psychiatric disorders	% MoBa	% All	Statistics
Affective disorders (F30–F39)	0.8	0.9	z = 0.42, *p* = 0.67
Anxiety disorders incl. OCD and SM (F40–F42, F94.0)	2.1	2.9	z = 1.90, *p* = 0.57
Adjustment disorders (F43)	1.2	2.3	z = 2.93, *p* = 0.03
Sleep disorders (F51)	0.6	0.5	z = 0.57, *p* = 0.57
Autism Spectrum Disorders (F84)	8.3	7.2	z = 1.70, *p* = 0.009
Conduct disorders (F91)	4.8	4.2	z = 1.20, *p* = 0.23
Mixed disorders of conduct and emotions (F92)	2.2	1.9	z = 0.88, *p* = 0.38
Emotional disorders with onset specific to childhood (F93)	3.6	4.3	z = 1.38, *p* = 0.17
Attachment disorders (F94.1-2)	1.5	2.7	z = 2.96, *p* = 0.003
Tics (F95)	8.5	7.5	z = 0.52, *p* = 0.13
Elimination disorders (F98)	3.1	2.6	z = 0.42, *p* = 0.21
Axis II, learning disabilities			
Specific disorders of speech and language (F80)	7.8	7.4	z = 0.61, *p* = 0.54
Specific disorder of scholastic skills (F81)	8.6	10.4	z = 2.36, *p* = 0.02
Specific disorder of motor function (F82)	2.8	2.1	z = 1.95, *p* = 0.05
Mixed specific developmental disorders (F83) ≥ 2 F80-F82)	2.4	2.4	z = 0, *p* = 1
Axis V, associated abnormal psychosocial situations	% MoBa	% All	
1 Abnormal intrafamilial relationships	6.6	8.7	z = 2.98, *p* = 0.003
2 Mental disorder, deviance or handicap in the child’s primary support group	14.4	17.0	z = 2.76, *p* = 0.005
3 Inadequate or distorted intrafamilial communication	2.4	3.1	z = 1.61, *p* = 0.11
4 Abnormal qualities of upbringing	2.6	3.3	z = 0.57, *p* = 0.12
5 Abnormal immediate environment	23.1	29	z = 5.19, *p* < 0.001
6 Acute life events	4.4	7.1	z = 4.20, *p* < 0.001
7 Societal stressors	0.8	2	z = 3.42, *p* = 0.006
8 Chronic interpersonal stress associated with school/work	4.1	5.1	z = 1.82, *p* = 0.07
9 Stressful events/situations resulting from the child’s own disorder/disability	5.1	5.8	z = 1.20, *p* = 0.23

*NPR* Norwegian Patient Registry; MoBa The Norwegian Mother and Child Cohort Study

## Abbreviations

ADHD: Attention deficit hyperactivity disorder
CAMHS: The Child and Adolescent Mental Health Services
CGAS: Children’s Global Assessment Scale
DSM-5: Diagnostic and statistical manual of mental disorders, 5^th^ edition
ICD-10: International classification of diseases and related health problems, 10th edition
MoBa: The Norwegian Mother and Child Cohort Study
NPR: Norwegian Patient Registry
